# In Vitro Photodynamic Effects of the Inclusion Nanocomplexes of Glucan and Chlorin e6 on Atherogenic Foam Cells

**DOI:** 10.3390/ijms22010177

**Published:** 2020-12-26

**Authors:** Jae Won Ahn, Jin Hyuk Kim, Kyeongsoon Park

**Affiliations:** Department of Systems Biotechnology, Chung-Ang University, Anseong, Gyeonggi 17546, Korea; pinkymonkey1@naver.com (J.W.A.); wlsgur0524@naver.com (J.H.K.)

**Keywords:** β-glucan, chlorin e6, dectin-1, photodynamic therapy, foam cells

## Abstract

Macrophage-derived foam cells play critical roles in the initiation and progression of atherosclerosis. Activated macrophages and foam cells are important biomarkers for targeted imaging and inflammatory disease therapy. Macrophages also express the dectin-1 receptor, which specifically recognizes β-glucan (Glu). Here, we prepared photoactivatable nanoagents (termed Glu/Ce6 nanocomplexes) by encapsulating hydrophobic chlorin e6 (Ce6) within the triple-helix structure of Glu in aqueous condition. Glu/Ce6 nanocomplexes generate singlet oxygen upon laser irradiation. The Glu/Ce6 nanocomplexes were internalized into foam cells and delivered Ce6 molecules into the cytoplasm of foam cells. Upon laser irradiation, they induced significant membrane damage and apoptosis of foam cells. These results suggest that Glu/Ce6 nanocomplexes can be a photoactivatable material for treating atherogenic foam cells.

## 1. Introduction

Atherosclerosis is a chronic inflammatory disease characterized by the accumulation of lipids in the large arteries. Various types of cells, such as endothelial cells, smooth muscle cells, and inflammatory cells are involved in the formation of atherosclerosis. Endothelial dysfunction initiates the entry of lipids and inflammatory cells into the large arteries [1,2,3,4]. In the artery, monocytes differentiate into macrophages, and the macrophages that take up the lipid become foam cells. These macrophage-derived foam cells are formed at early stages in the atherosclerotic lesions and play critical roles in the development of atherosclerosis [5,6]. Macrophages express a variety of surface recognition receptors that serve as target biomarkers for imaging and therapy [7,8,9,10,11,12]. Dectin-1, a specific receptor for β-glucan, is one such receptor expressed on macrophages [13], including plaque macrophages of atherosclerotic mice [14]. Therefore, the specific targeting of the dectin-1 receptor on foam cells is an alternative approach for imaging and treatment of atherosclerosis.

Photodynamic therapy (PDT) is an effective therapeutic strategy for cancer. The use of photosensitizers and a specific wavelength of light generate reactive oxygen species (ROS), such as singlet oxygen (SO), hydroxyl radicals, and superoxide that subsequently induce membrane damage in the target cells, leading to cell death [15,16]. PDT is also used as an alternative therapy for atherosclerosis [17,18]. Previous studies have shown that PDT promotes plaque stabilization in atherosclerotic lesions and inhibits plaque progression by reducing the macrophage content [19,20,21,22,23]. Among various photosensitizers, chlorin e6 (Ce6) is widely used for PDT because it has high photosensitizing effect, low dark toxicity, long absorption band (663 nm), deep tissue penetration, and higher production of singlet oxygen [24]. However, despite the success of these PSs for PDT in in vitro and in vivo studies, some issues such as its insolubility in water and low selectivity to target cells or tissues still need to be overcome [25,26]. 

β-Glucan is a natural polysaccharide comprising linear β-1,3-linked D-glucose molecules, and possessing anti-inflammatory, anti-oxidant, and antifungal activities [13,27,28]. Glu acts as a ligand for the dectin-1 receptor and complement receptor 3 [27] on macrophages and dendritic cells in the immune system. As the uptake of Glu molecules is dectin-1-dependent in vitro [29], the selective targeting of dectin-1 makes the Glu nanoparticles an attractive drug carrier for the delivery of proteins [29], nucleic acids such as DNA [30] and siRNA [31], and small drug molecules [32].

In this study, we developed new photoactivatable nanoagents (termed Glu/Ce6 nanocomplexes) to deliver Ce6 into foam cells and enhance the effects of PDT on foam cells after laser irradiation. These nanoagents were prepared by encapsulating hydrophobic Ce6 within the triple-helix structure of Glu molecules. We analyzed their physicochemical and optical properties, as well as their ability to generate SO after laser treatment. We evaluated their intracellular uptake into foam cells to check whether Glu/Ce6 nanocomplexes can deliver Ce6 into the foam cells. We also confirmed their effects on foam cells after laser irradiation. 

## 2. Results and Discussion

### 2.1. Synthesis of Inclusion Nanocomplexes of Glucan and Chlorin e6 (Glu/Ce6) 

**Figure 1:**
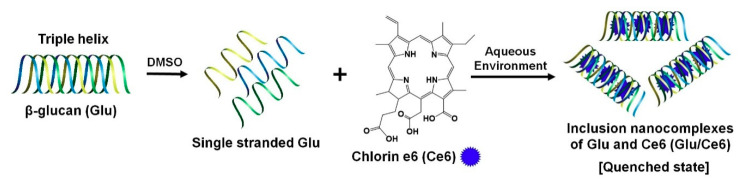
Schematic illustration of the preparation procedures of the Glu/Ce6 nanocomplexes.

Typically, Glu has triple-helix structures in aqueous solution, which change into single strands upon solubilization at temperatures over 150 °C, or with NaOH or dimethyl sulfoxide (DMSO). Interestingly, they can re-assemble the triple-helix structure when exposed to an aqueous environment again [33,34]. Based on this fact, we prepared photoactivatable Glu/Ce6 nanocomplexes. For the synthesis of Glu/Ce6 nanocomplexes, Glu was dissolved in DMSO with heating at 60 °C to release single spiral structures from triple-spiral structures due to the loss of hydrophilicity and hydrogen bonds [35]. Then, hydrophobic Ce6 was added to the Glu solution and stirred for 3 h. The mixture was then added into a large amount of water. On exposure to an aqueous environment, the Glu triple structures may be reformed with the Ce6 molecules loaded into them to form Glu/Ce6 inclusion nanocomplexes (Figure 1).

### 2.2. Characterizations of Glu/Ce6 Nanocomplexes

**Figure 2:**
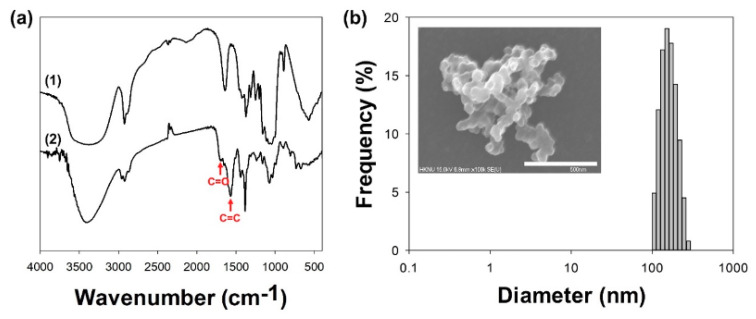
Characterizations of Glu/Ce6 nanocomplexes. (**a**) FTIR spectra of (1) Glu and (2) Glu/Ce6. (**b**) Particle size distribution of Glu/Ce6. Inset SEM image: the morphology of Glu/Ce6. Scale bar: 500 nm.

After synthesis and purification of the Glu/Ce6 nanocomplexes, the loading amount of Ce6 within the nanocomplexes was determined by performing ultraviolet/visible (UV/Vis) analysis. Based on the standard curve of Ce6, it was determined that 1 mL of Glu/Ce6 nanocomplex solution contained 1.652 mM of Ce6. The synthesis of Glu/Ce6 nanocomplexes was further confirmed by Fourier-transform infrared (FTIR) analysis (Figure 2a). After loading of Ce6 into Glu, two new peak bands were observed at 1698 and 1570 cm^−1^ for the C = O and C = C stretching vibrations of Ce6 molecules, respectively.

The dissolved Glu in DMSO has a single stranded structure, but it is converted into the triple-helix structure (known as a renaturation process) when exposed to water (at a ratio of 20 or higher) or when granulated to a size of ~ 800 nm [35]. In this study, the determined particle mean sizes of granulated Glu were 488 ± 35.6 nm (Supplementary Materials Figure S1), but polydispersity index (PDI) and Z-average sizes were not detected, suggesting that granulated Glu has the multi-disperse particle structures. After loading of Ce6 into Glu molecules, the mean sizes of the Glu/Ce6 nanocomplexes were 153.8 ± 35.6 nm, their Z-average sizes were 169.0 nm, with a PDI of 0.343 and a spherical shape (Figure 2b). SEM image showed that Glu/Ce6 nanocomplexes formed the multimeric or aggregated structures in dried state. Compared to the sizes of granulated Glu, Glu/Ce6 nanocomplexes were much smaller, due to stronger intermolecular hydrophobic interactions between Ce6 and Glu within the nanocomplexes.

**Figure 3:**
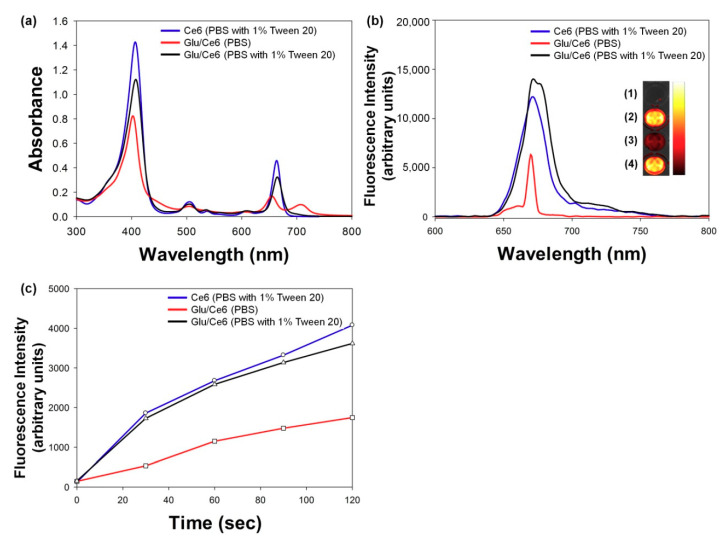
Optical characterizations of Glu/Ce6 and singlet oxygen generation by Glu/Ce6 upon laser irradiation. (**a**) UV/Vis and (**b**) fluorescence spectra of Ce6 (PBS with 1% Tween 20), Glu/Ce6 (PBS only or PBS with 1% Tween 20). Inset: Fluorescence image of (1) PBS only, (2) Ce6 (PBS with 1% Tween 20), (3) Glu/Ce6 (PBS), and (4) Glu/Ce6 (PBS with 1% Tween 20). (**c**) Singlet oxygen generation from Ce6 (PBS with 1% Tween 20), Glu/Ce6 (PBS), and Glu/Ce6 (PBS with 1% Tween 20), obtained by monitoring the fluorescence intensity (FI) of SOSG at 525 nm at different times of exposure to 670 nm laser.

Optical properties were examined by using UV/Vis and fluorescence spectrophotometers to demonstrate whether Glu/Ce6 re-assembled its triple-helix structure in aqueous solution. The solubilized Ce6 and Glu/Ce6 nanocomplexes in PBS (pH 7.4) solution with 1% Tween 20 (surfactant) showed a strong and sharp Soret band at 405 nm and Q-bands ranging from 480 to 700 nm. However, these peaks were relatively lower and broadened when Glu/Ce6 nanocomplexes were exposed to PBS (pH 7.4) without Tween 20 due to the interference of light transmittance (Figure 3a). In line with this result, fluorescence analyses demonstrated that the solubilized Ce6 and Glu/Ce6 in PBS (pH 7.4) with 1% Tween 20 showed strong fluorescence intensities, whereas Glu/Ce6 nanocomplexes dispersed in PBS without Tween 20 exhibited weak fluorescence intensities owing to the fluorescence quenching mechanism between Ce6 molecules within the Glu/Ce6 nanocomplexes (Figure 3b). These results suggested that the fabricated Glu/Ce6 re-assembled its triple-helix structure by a renaturation process due to intermolecular hydrophobic interactions between Ce6 and Glu molecules.

### 2.3. Singlet Oxygen (SO) Generation Study

Photoactivatable nanomaterials should generate SO upon laser irradiation for PDT applications. To demonstrate whether Glu/Ce6 nanocomplexes can generate SO upon laser irradiation, a singlet oxygen sensor green (SOSG) study was performed because it is highly selective for ^1^O_2_ [36]. Figure 3c shows the changes in SOSG fluorescence intensity with laser irradiation time. Ce6 and Glu/Ce6 solubilized in PBS (pH 7.4) containing 1% Tween 20 increased SOSG fluorescence signals with increasing irradiation time, whereas Glu/Ce6 nanocomplexes dispersed in PBS without a surfactant showed comparatively decreased fluorescence signals. As seen in UV/Vis and fluorescence analyses, the nanoparticle state (quenched state) of Glu/Ce6 has a relatively low absorbance and fluorescence intensity. Based on these results, we hypothesized that Glu/Ce6 nanocomplexes dispersed in PBS without a surfactant generate relatively lower levels of SO upon laser irradiation compared to the solubilized Ce6 and Glu/Ce6.

### 2.4. In Vitro Cytotoxic Study of Glu/Ce6 Nanocomplexes on Macrophages and Foam Cells 

**Figure 4:**
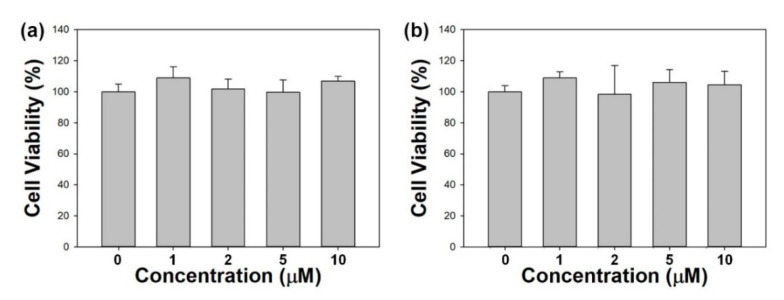
In vitro cytotoxic effects of Glu/Ce6 on (**a**) normal macrophages and (**b**) foam cells.

In vitro cytotoxicity of the Glu/Ce6 nanocomplexes was evaluated in normal macrophages and foam cells. Various concentrations of Glu/Ce6 (equivalent of 1, 2, 5, and 10 µM Ce6) were allowed to react with macrophages and foam cells in the dark. Figure 4a,b shows that macrophages and foam cells maintained their cell viabilities above 95% at all tested concentrations, suggesting that Glu/Ce6 nanocomplexes did not induce any cellular toxicity.

### 2.5. Intracellular Uptake of Glu/Ce6 Nanocomplexes by Foam Cells 

**Figure 5:**
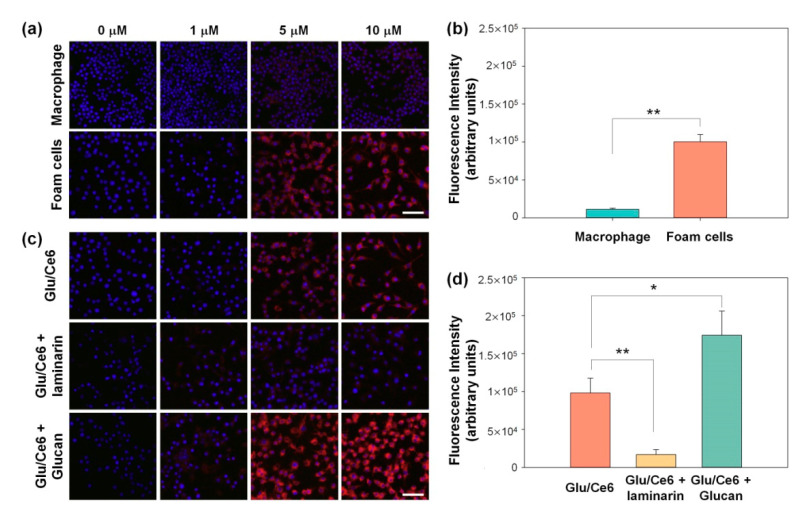
In vitro cellular internalization of Glu/Ce6 into macrophages and foam cells. (**a**) Confocal microscopy images of Glu/Ce6 internalization into macrophages and foam cells. Scale bar: 50 µm. (**b**) Comparison of cellular internalization of Glu/Ce6 (equivalent 5 μM Ce6) into normal macrophages and foam cells. (**c**) Cellular uptake of Glu/Ce6 against foam cells with or without pretreatment with laminarin or β-glucan. Scale bar: 50 µm. (**d**) Comparison of Glu/Ce6 (equivalent 5 μM Ce6) cellular uptake against foam cells with or without laminarin or β-glucan. ** *p* < 0.01, * *p* < 0.05.

Glu is a specific ligand for the dectin-1 receptor, which is expressed on macrophages [13]. We investigated whether Glu/Ce6 nanocomplexes are taken up by both macrophages and foam cells, using a customized multi-channel confocal laser scanning microscope (CLSM). Figure 5a shows that the intracellular uptake of Glu/Ce6 nanocomplexes by foam cells increased with increasing doses and was approximately nine-fold higher compared to macrophages (Figure 5b), suggesting that Glu/Ce6 nanocomplexes can deliver Ce6 molecules into foam cells with a better efficacy than normal macrophages. 

Next, we performed blocking experiments by pretreating the foam cells with free laminarin (soluble β-glucan for dectin-1 ligand) and free Glu to confirm whether the cellular uptake of Glu/Ce6 nanocomplexes occurs via dectin-1 receptor-mediated endocytosis. The pretreatment of foam cells with free laminarin led to a significant decrease (of up to 7.15-fold) in the cellular uptake of Glu/Ce6 nanocomplexes because laminarin blocks the binding of foam cells to dectin-1 of Glu/Ce6 nanocomplexes (Figure 5c,d) [37,38]. In contrast, pretreatment with free Glu increased the cellular uptake of Glu/Ce6 nanocomplexes up to approximately 1.75-fold (Figure 5c,d). This increase might be associated with the increase of dectin-1 protein in macrophages treated with β-glucan [39]. Although we have limitation for dectin-1 binding affinity of Glu/Ce6 nanocomplexes on foam cells, these results suggest that Glu/Ce6 nanocomplexes deliver Ce6 molecules into foam cells with a better efficacy compared to normal macrophages.

### 2.6. In Vitro Phototoxic Effects of Glu/Ce6 Nanocomplexes

**Figure 6:**
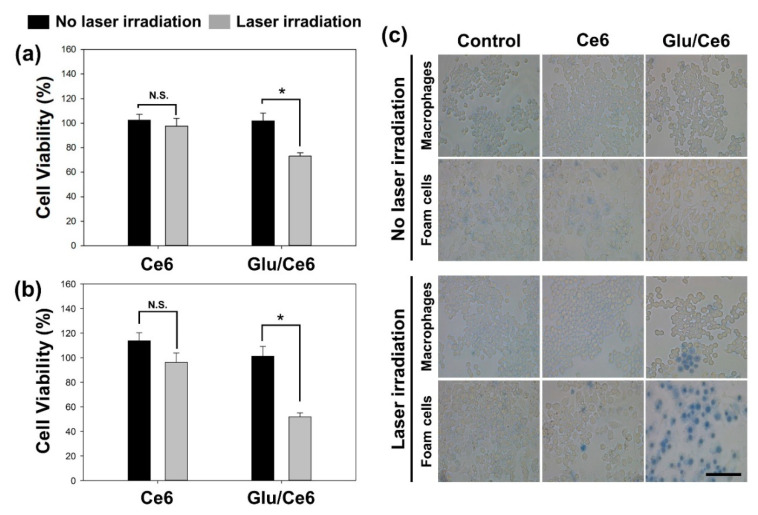
In vitro phototoxic effects of Ce6 and Glu/Ce6 on macrophages and foam cells. Comparison of in vitro phototoxic effects of Ce6 (2 μM) and Glu/Ce6 (equivalent 2 μM Ce6) on (**a**) macrophages and (**b**) foam cells before and after laser irradiation (670 nm, 1 min irradiation at 50 mW/cm^2^). N.S.: Not Significant. * *p* < 0.05. (**c**) Trypan blue staining of macrophages and foam cells treated with Ce6 (2 μM Ce6) and Glu/Ce6 (equivalent 2 μM Ce6) before and after laser irradiation. Scale bar: 100 µm.

In vitro phototoxic effects of Glu/Ce6 nanocomplexes on macrophages or foam cells were first evaluated by the cell counting kit-8 (CCK-8) assay. Cell viability was normalized to control cells (no drug treatment, but laser irradiation). Macrophages or foam cells were not damaged by Glu/Ce6 nanocomplexes under dark conditions, as shown in Figure 4. In addition, after these cells were treated with free Ce6 (2 μM), their cell viabilities were above 95% and almost all cells were viable after NIR laser irradiation (Figure 6a,b). However, the cells treated with Glu/Ce6 nanocomplexes (equivalent 2 μM Ce6) were photodamaged after laser irradiation, and the viabilities of macrophages and foam cells were 73.1% and 51.8%, respectively. 

To confirm the in vitro PDT effects of Glu/Ce6 nanocomplexes, we performed a trypan blue dye exclusion test, which is a widely used test to identify dead cells. The live cells with intact membranes remain unstained, whereas dead cells with compromised membranes become stained [40]. As shown in Figure 6c, macrophages and foam cells treated with free Ce6 or Glu/Ce6 without laser irradiation were not stained, implying that their membranes were intact. Additionally, although some of the foam cells treated with both free Ce6 and NIR laser were stained, most of the cell membranes were not photo-damaged. However, when both macrophages and foam cells were treated with Glu/Ce6, as well as laser irradiation, the number of trypan blue-stained cells increased, indicating that the membranes of foam cells were more photo-damaged, as compared to those of macrophages.

**Figure 7:**
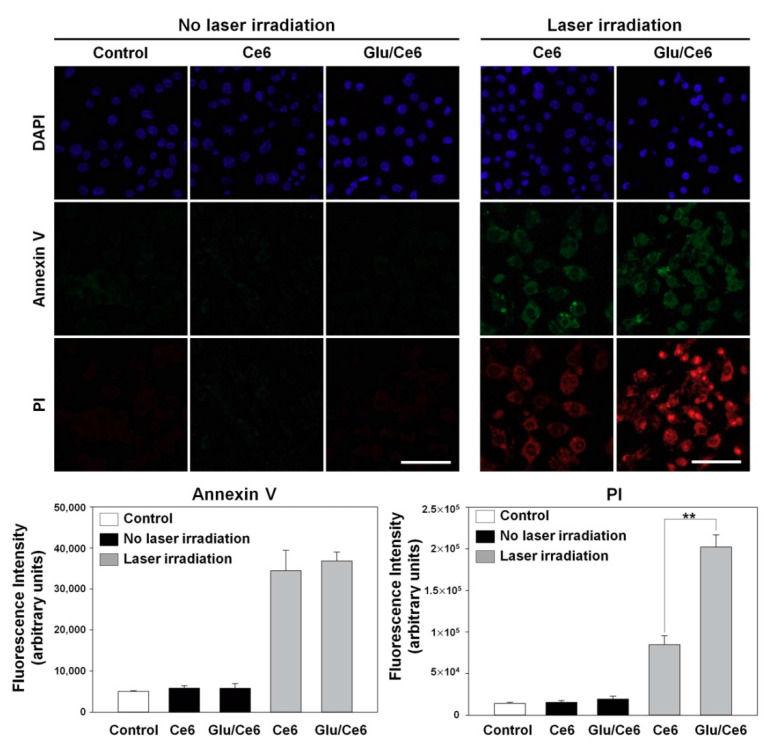
In vitro phototoxic effects of Ce6 and Glu/Ce6 on foam cells before and after laser irradiation (670 nm, 1 min irradiation at 50 mW/cm^2^). (**a**) Annexin V and propidium iodide (PI) stainings were done to evaluate PDT effects of Ce6 and Glu/Ce6 on foam cells. Scale bar: 30 µm. Quantitative fluorescence intensity (FI) of (**b**) annexin V (AV) and (**c**) propidium iodide (PI) for each experimental group. ** *p* < 0.01.

Photoactivatable nanoagents induce cell apoptosis after laser treatment and then exert PDT effects. To demonstrate whether Glu/Ce6 nanocomplexes induce the apoptosis of foam cells compared to free Ce6, an annexin V (AV)/propidium iodide (PI) stain experiment was performed. The AV/PI staining method is useful for monitoring the progression of cell apoptosis because the early apoptotic cells are stained with AV but not PI, and late apoptotic cells are positively stained with both AV and PI [41]. Without NIR laser application, the control cells, free Ce6-treated and Glu/Ce6-treated foam cells did not show fluorescence signals (Figure 7a). After laser irradiation, both free Ce6-treated and Glu/Ce6-treated foam cells displayed similar AV-fluorescence signals in the representative fluorescence images (Figure 7a) and the quantitative comparison result of AV-fluorescence signals in both groups supported the fluorescence images (Figure 7b). However, Glu/Ce6-treated foam cells had significantly higher PI fluorescence intensities than free Ce6-treated foam cells (Figure 7a,c), suggesting that Glu/Ce6 nanocomplexes exhibit stronger PDT effects on foam cells compared to free Ce6. This enhanced PDT effect of Glu/Ce6 nanocomplexes on foam cells is associated with higher intracellular internalization compared to free Ce6. Indeed, Glu/Ce6 nanocomplexes showed strong fluorescence signals in the cytoplasm of foam cells and their intracellular uptake increased up to 2.6-fold, compared to free Ce6 (Supplementary Materials Figure S2), which in turn led to enhanced PDT effects. Thus, the delivery of Ce6 molecules into foam cells, using Glu/Ce6 nanocomplexes improves the light-triggered PDT effects.

## 3. Materials and Methods

### 3.1. Materials

Glu (β-1,3-Glucan from *Euglena gracilis*), potassium bromide (KBr), and LPS were purchased from Sigma-Aldrich (St. Louis, MO, USA). DMSO was obtained from Duksan (Ansan, Korea). A dialysis membrane (MWCO: 12–14 kDa) was purchased from Spectrum Laboratories (Rancho Dominguez, CA, USA). Ce6 and SOSG were procured from Frontier Scientific (Logan, UT, USA) and Invitrogen (Carlsbad, CA, USA), respectively.

### 3.2. Fabrication of Glu and Ce6 (Glu/Ce6) Inclusion Nanocomplexes

To fabricate the Glu/Ce6 nanocomplexes, 40 mg of Glu was added to 10 mL of DMSO and dissolved by heating at 90 °C overnight. Then, 20 mg of Ce6 dissolved in DMSO (1 mL) was added to the Glu solution and stirred for 3 h. Afterwards, the mixture was added to 200 mL of ultrapure water at 2 mL/min, using a syringe pump (NE-300, New Era Pump Systems Inc., Farmingdale, NY, USA) and stirred for 3 h. The resulting mixture was then dialyzed against deionized (DI) water for 3 days, using a dialysis membrane (MWCO: 12–14 kDa). Finally, the dialyzed mixture was centrifuged at 879× *g* for 10 min to remove large particles, and the supernatant was carefully collected and concentrated, using a rotary evaporator (N-1200BS, EYELA, Bohemia, NY, USA). Concentrated Glu/Ce6 nanocomplexes in DI water were used for the in vitro experiments. For FTIR analysis, a powder of Glu/Ce6 was obtained by freeze-drying the concentrated Glu/Ce6 nanocomplexes for 3 days. 

### 3.3. Characterizations of Glu/Ce6 Nanocomplexes

To determine inclusion efficiency (loading efficiency) of Ce6 in Glu/Ce6, the concentrated Glu/Ce6 (1 mL) was added to PBS (1 mL) containing 1% Tween 20, and the mixed solution was sequentially diluted, using 1% Tween-containing PBS to measure the absorbance of Ce6. The amount of Ce6 in Glu/Ce6 was calculated, using the following equation, which was obtained from the standard curve of Ce6 (Y = 243.46x + 0.0018 (R^2^ = 1)) by measuring the absorbance at 407 nm with a UV/Vis spectrophotometer (Neo-S450, Neogen, Korea).

The obtained Glu/Ce6 powder was further characterized by Fourier transform infrared (FTIR, Shimadzu 8400S, Kyoto, Japan) spectroscopy. The FTIR spectrum was acquired by using the KBr pellet method at a resolution of 4000–400 cm^–1^.

The particle sizes and distribution of Glu/Ce6 were determined as follows: A 10-fold diluted Glu/Ce6 solution was prepared by using DI water. Then, the particle size, Z-average size, and polydispersity index were determined at a scattering angle of 90° with a particle size analyzer (SZ-100, HORIBA, Kyoto, Japan). To observe the morphology, a drop of a 10-fold diluted Glu/Ce6 solution was added to a cover slip and air-dried. Then, after platinum coating, the morphology of the dried Glu/Ce6 nanocomplexes was analyzed by using a field-emission SEM (FE-SEM, Baltec S-4700, Hitachi, Tokyo, Japan).

To confirm whether the prepared Glu/Ce6 forms nanosized particles, UV/Vis absorbance (from 300 to 800 nm) and fluorescence spectra (from 600 to 800 nm) of Glu/Ce6 (equivalent 5 μM Ce6) diluted in PBS (pH 7.4) were recorded with a UV/Vis spectrophotometer (Neo-S450, Neogen, Sejong, Korea) and fluorescence spectrometer (FS-2, Scinco, Korea), respectively. For comparison, free Ce6 (5 μM) and Glu/Ce6 (equivalent 5 μM Ce6) solutions were prepared, using 1% Tween 20-containing PBS, and their UV/Vis absorbance and fluorescence spectra were also monitored. In addition, the fluorescence images of these three solutions were acquired by using a small animal imaging equipment (In Vivo Smart-LF, VIEWORKS, Anyang, Korea).

### 3.4. Singlet-Oxygen-Generation Study

The SO generation potency of Glu/Ce6 was investigated by monitoring the changes in SOSG fluorescence intensity upon laser irradiation (670 nm, irradiation dose rate: 50 mW/cm^2^). Oxygen-saturated PBS (pH 7.4) was prepared by bubbling oxygen gas for 30 min. Three solutions –Glu/Ce6 (equivalent 5 μM Ce6) in O_2_-saturated PBS (pH 7.4) without Tween 20, Glu/Ce6 (equivalent 5 μM Ce6) in O_2_-saturated PBS containing 1% Tween 20, and Ce6 (5 μM) in O_2_-saturated PBS containing 1% Tween 20, were prepared. These three solutions contained 1 µM of the SOSG reagent. Under different exposure times from 0 to 120 sec with 670 nm laser irradiation, the fluorescence intensities of SOSG (Ex/Em: 504 nm/525 nm) were recorded with a fluorescence spectrometer (FS-2, Scinco, Korea).

### 3.5. Cells and Culture Condition

Murine macrophages (RAW 264.7) were obtained from the Korean Cell Line Bank (KCLB, Seoul, Korea). Cells were maintained in Dulbecco’s Modified Eagle Medium (DMEM, Welgene, Seoul, Korea) containing 10% heat-inactivated FBS and 1% penicillin-streptomycin at 37 °C in a humidified 5% CO_2_ incubator.

### 3.6. Cytocompatibility Study

In vitro cytocompatibility of the Glu/Ce6 on macrophages and atherogenic foam cells was evaluated by using the CCK-8 assay kit (Dojindo, Kumamoto, Japan) according to the manufacturer’s instructions. Macrophages (1 × 10^4^ cells/well) were seeded into 96-well plates and incubated for 24 h. To prepare atherogenic foam cells, the cultured macrophages were treated with LDL (100 µg/mL) and LPS (200 ng/mL) for 24 h. The cells were then treated with various concentrations of Glu/Ce6 (equivalent of 1, 2, 5, and 10 µM Ce6) for 24 h. After carefully washing the cells with culture medium, CCK-8 reagent solution was added to each well and further incubated for 2 h. The absorbance was measured at 450 nm, using Multiskan Go (Thermo Fisher Scientific, MA, USA). Cell viability was expressed as the percentage of viable cells compared to the survival of the non-treated control group. 

### 3.7. Intracellular Uptake of Glu/Ce6 

To compare the intracellular internalization ability of free Ce6 and Glu/Ce6 into normal macrophages and atherogenic foam cells, macrophages (1 × 10^5^ cells/mL) incubated into 4-well chamber slides. Atherogenic foam cells were prepared by treating the prepared normal macrophages with LPS and LDL for 24 h. The two kinds of cells were treated with various concentrations of free Ce6 (1, 5, and 10 µM Ce6) and Glu/Ce6 (equivalent of 1, 5, and 10 µM Ce6) for 2 h. The cells were then carefully washed with PBS (pH 7.0) and fixed with formalin for 30 min. The nuclei of the cells were counterstained, using fluoromount solution containing 4*′*, 6*′*-diamidino-2-phenylindole hydrochloride (DAPI) (SouthernBiotech, AL, USA). To further evaluate whether Glu/Ce6 nanocomplexes are internalized into atherogenic foam cells, the foam cells were prepared by using the same protocols as described above. After pretreating the cells with free laminarin (1 mg/mL) as a soluble dectin-1 ligand or free β-glucan (1 mg/mL) as a dectin-1 receptor activator, the cells were treated with various concentrations of Glu/Ce6 (equivalent of 1, 5, and 10 µM Ce6) for 2 h. Afterwards, the cells were washed and fixed with formalin. The cell nuclei were counterstained with DAPI. The intracellular uptake of Glu/Ce6 into the cells was observed with a customized multi-channel confocal laser scanning fluorescence microscope.

Moreover, to quantitatively compare the intracellular uptake of free Ce6 and Glu/Ce6 in the absence and presence of laminarin or Glu, the average fluorescence intensity of a single cell was quantified by using Image J software (Ver 1.53a, National Institutes of Health, MD, USA). This was done by dividing the number of selected cells (approximately 50 cells) by the total fluorescence intensity of the selected cells from the confocal images. 

### 3.8. In Vitro Phototoxic Effects of Glu/Ce6 

To investigate whether Glu/Ce6 has phototoxic effects on normal macrophages or atherogenic foam cells under laser irradiation, CCK-8 assay, trypan blue dye exclusion, and AV/PI staining were performed. For the CCK-8 assay, the prepared normal macrophages and foam cells were treated with Ce6 (2 µM) or Glu/Ce6 (equivalent 2 µM Ce6). After 2 h of incubation, the culture medium was freshly changed and the cells were irradiated with the NIR laser (670 nm CW laser, 50 mW/cm^2^) for 1 min. The laser power density and irradiation time were chosen based on our previous study [21]. They were then treated with CCK-8 solution (10 µL) and further incubated for 1 h. Phototoxic effects of each group were analyzed by measuring the absorbance at 450 nm, using a microplate reader (Thermo Fisher Scientific, Waltham, MA, USA), and the cell viability was represented as the percentage of viable cells compared to that of non-treated control cells.

For the trypan blue dye exclusion test, normal macrophages or foam cells were treated with free Ce6 or Glu/Ce6 at the same concentration as described above for 2 h. After the cells were carefully washed with fresh culture medium, they were irradiated with an NIR laser (670 nm CW laser, 50 mW/cm^2^) for 1 min. Next, the cells were treated with trypan blue solution for 30 min and the excess trypan blue solution was then removed. After carefully adding PBS (pH 7.4) into each cell, optical cell images were acquired under a light microscope at 20× magnification, using LEICA DMi1 (Leica Microsystems, Wetzlar, Germany).

To evaluate apoptosis induction after laser irradiation, macrophages (1 × 10^5^ cells/mL) were seeded into a 4-well chamber and allowed to adhere for 24 h. Atherogenic foam cells were prepared by treating LPS and LDL for 24 h as described above. The cells were exposed to free Ce6 (5 µM) or Glu/Ce6 (equivalent of 5 µM Ce6) for 2 h. After carefully washing with PBS, they were irradiated with or without an NIR laser (670 nm, 50 mW/cm^2^) for 1 min. Then, the cells were stained with AV/PI for 5 min according to the manufacturer’s instructions. Next, the cells were washed with the 1× binding buffer, fixed with paraformaldehyde (4%) for 30 min, and washed with PBS. The nuclei of the cells were counterstained with a mounting solution containing DAPI. Apoptosis induction was observed under a confocal microscope (LSM 700, Carl Zeiss, Germany). 

### 3.9. Statistical Analysis

Statistical analysis was performed by using the R software (v3.4.3, Boston, MA, USA). Data are expressed as the mean ± SD. Statistical comparisons between two groups were carried out by using the Mann–Whitney test. Statistical significance was represented by *p*-values less than 0.01 or 0.05. 

## 4. Conclusions

We fabricated Glu/Ce6 nanocomplexes as photoactivatable agents by encapsulating Ce6 molecules within Glu with a triple-helix structure, in aqueous conditions. The Glu/Ce6 nanocomplexes generated SO after NIR laser irradiation. Cytocompatible Glu/Ce6 nanocomplexes efficiently delivered Ce6 molecules into foam cells, as compared to normal macrophages. Without laser irradiation, the Glu/Ce6 nanocomplexes did not induce cell death. However, laser treatment significantly damaged the foam cell membranes and increased cell apoptosis. In the present study, we demonstrated the in vitro PDT effects of Glu/Ce6 nanocomplexes on foam cells. Unfortunately, the storage stability issue of Glu/Ce6 nanocomplexes should be improved because the prepared Glu/Ce6 nanocomplexes formed much bigger aggregates during the storage. In the near future, we will improve the colloidal stability of Glu/Ce6 nanocomplexes by modifying them and perform further PDT study to demonstrate whether Glu/Ce6 nanocomplexes are effective in regressing or stabilizing atherosclerotic plaques in vivo.

## Supplementary Materials

The following are available online at https://www.mdpi.com/1422-0067/22/1/177/s1, Figure S1: Particle size distribution of granulated Glu, Figure S2: Intracellular uptake of free Ce6 and Glu/Ce6 against foam cells.
