# Supplementary material for: In Vitro Photodynamic Effects of the Inclusion Nanocomplexes of Glucan and Chlorin e6 on Atherogenic Foam Cells
Source: Int J Mol Sci. 2020 Dec 26;22(1):177. doi: 10.3390/ijms22010177 (PMC7795021; doi:10.3390/ijms22010177)
Supplement: Supplementary file 1 [file ijms-22-00177-s001.pdf]

Supplementary Materials

In Vitro Photodynamic Effects of the Inclusion Nanocomplexes of Glucan and Chlorin e6 on Atherogenic Foam Cells

Jae Won Ahn†, Jin Hyuk Kim† and Kyeongsoon Park*

Department of Systems Biotechnology, Chung-Ang University, Anseong, Gyeonggi 17546, Korea; pinkymonkey1@naver.com (J.W.A); wlsgur0524@naver.com (J.H.K)
* Correspondence: kspark1223@cau.ac.kr; Tel.: +82-031-670-3357
† These authors contributed equally to this work.

Particle sizes of granulated Glu

To prepare granulated Glu, 1 mg of Glu was dissolved in DMSO (0.4 mL) by vortexing for 10 min. Glu solution was added drop-by-drop into 1 mL of deionized water and sonicated for 5 min. Then, the particle sizes of granulated Glu were analyzed with a particle size analyzer (SZ-100, HORIBA, Kyoto, Japan). The determined mean sizes of granulated Glu were 488 ± 75.6 nm. But, polydispersity index and Z-average size of granulated Glu were not determined.

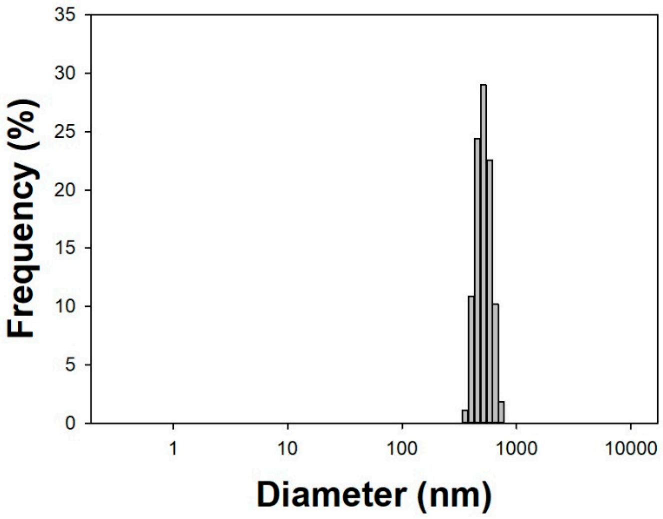

Figure S1. Particle size distribution of granulated Glu.

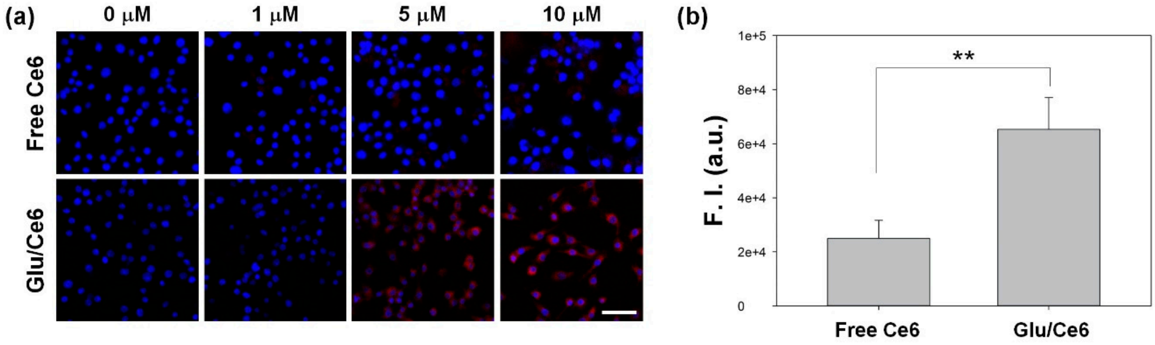

Figure S2. (a) Intracellular uptake of free Ce6 and Glu/Ce6 against foam cells. Scale bar: 50 μm. (b) Quantitative comparison of cellular uptake between free Ce6 (equiv. 5 μM Ce6) and Glu/Ce6 (equiv. 5 μM Ce6) against atherogenic foam cells. $^{**}P < 0.01$
